# Supplementary material for: Illuminating the druggable genome through patent bioactivity data
Source: PeerJ. 2023 May 2;11:e15153. doi: 10.7717/peerj.15153 (PMC10162037; doi:10.7717/peerj.15153)
Supplement: Supplemental Information 1 — *TDL: target development level at the start of this work. [file peerj-11-15153-s001.docx]

| IDG Family | TDL* | Target ID | Target Name | Target type | Accession | Patents |
| --- | --- | --- | --- | --- | --- | --- |
| Other | Tbio | CHEMBL1741179 | DNA dC->dU-editing enzyme APOBEC-3A | Single protein | P31941 | WO-2015106272-A1 |
| Enzyme | Tbio | CHEMBL4523467 | Acetyl-coenzyme A synthetase, cytoplasmic | Single protein | Q9NR19 | US-20170088523-A1 |
| Enzyme | Tbio | CHEMBL4105941 | 2-amino-3-carboxymuconate-6-semialdehyde decarboxylase | Single protein | Q8TDX5 | US-20180134667-A1  US-9708272-B2 |
| Enzyme | Tbio | CHEMBL3886062 | Cytosolic carboxypeptidase 2 | Single protein | Q5U5Z8 | US-9199918-B2 |
| Enzyme | Tbio | CHEMBL3257 | Aldehyde oxidase | Single protein | Q06278 | WO-2018198842-A1 |
| Other | Tclin Tbio | CHEMBL2095226 | Alpha5beta1 | Protein complex | P05556  P08648 | US-8501787-B2 |
| Other | Tbio | CHEMBL1741217 | DNA dC->dU-editing enzyme APOBEC-3G | Single protein | Q9HC16 | WO-2015106272-A1 |
| Other | Tbio | CHEMBL4523224 | Aquaporin-2 | Single protein | P41181 | US-20160264604-A1 |
| Other | Tbio | CHEMBL5964 | Aquaporin-4 | Single protein | P55087 | US-20160264604-A1 |
| Epigenetic | Tbio | CHEMBL3588739 | Histone-lysine N-methyltransferase ASH1L | Single protein | Q9NR48 | WO-2017197240-A1 |
| Enzyme | Tbio | CHEMBL1741221 | Cysteine protease ATG4B | Single protein | Q9Y4P1 | WO-2017027984-A1  WO-2018096088-A1 |
| Other | Tbio | CHEMBL2321621 | Ubiquitin-like modifier-activating enzyme ATG7 | Single protein | O95352 | WO-2018089786-A1 |
| Transcription Factor | Tbio | CHEMBL4295651 | Transcription regulator protein BACH1 | Single protein | O14867 | US-20170231967-A1 |
| Other | Tbio | CHEMBL4523197 | B-cell lymphoma 3 protein | Single protein | P20749 | US-20160185740-A1 |
| Other | Tbio | CHEMBL3712821 | B-cell CLL/lymphoma 9 protein | Single protein | O00512 | US-20180092866-A1 |
| Transcription Factor | Tbio | CHEMBL1250348 | Myc proto-oncogene protein | Single protein | P01106 | US-20140296307-A1  US-20150291521-A1  US-20160264560-A1  US-20170233405-A1  US-20180162851-A1  US-20180291035-A1  US-20180354972-A1  US-9387213-B2  WO-2016115360-A1  WO-2018112545-A1 |
| GPCR | Tbio | CHEMBL4523478 | C5a anaphylatoxin chemotactic receptor 2 | Single protein | Q9P296 | US-20170129921-A1 |
| Other | Tbio | CHEMBL4523126 | Calpain-9 | Single protein | O14815 | US-20180318405-A1 |
| Enzyme | Tbio | CHEMBL4105728 | Cyclic GMP-AMP synthase | Single protein | Q8N884 | WO-2017176812-A1 |
| Enzyme | Tbio | CHEMBL3797014 | ATP-dependent Clp protease ATP-binding subunit clpX-like, mitochondrial | Single protein | O76031 | WO-2018114965-A1 |
| Enzyme | Tbio | CHEMBL4296242 | Connector enhancer of kinase suppressor of ras 1 | Single protein | Q969H4 | EP-2931280-B1  US-20160304533-A1 |
| Other | Tbio | CHEMBL5661 | Exportin-1 | Single protein | O14980 | US-20130281389-A1 |
| Enzyme | Tbio | CHEMBL2146 | NADH-cytochrome b5 reductase 3 | Single protein | P00387 | WO-2016040803-A1 |
| Other | Tbio | CHEMBL3542436 | cytochrome p450 2a13 | Single protein | Q16696 | US-8598165-B2  US-8609708-B2 |
| Enzyme | Tbio | CHEMBL4523510 | Cholesterol 24-hydroxylase | Single protein | Q9Y6A2 | US-9643957-B2  US-20170182047-A9 |
| Enzyme | Tbio | CHEMBL4523494 | 7-alpha-hydroxycholest-4-en-3-one 12-alpha-hydroxylase | Single protein | Q9UNU6 | WO-2014018375-A1  WO-2018034917-A1 |
| Enzyme | Tbio | CHEMBL2311243 | d-3-phosphoglycerate dehydrogenase | Single protein | O43175 | WO-2018076537-A1  WO-2018167019-A1 |
| Other | Tbio | CHEMBL4523330 | Disabled homolog 2-interacting protein | Single protein | Q5VWQ8 | US-20170304392-A1 |
| Other | Tbio | CHEMBL4105866 | DEP domain-containing mTOR-interacting protein | Single protein | Q8TB45 | WO-2018085753-A1 |
| Other | Tbio | CHEMBL4523267 | Dermcidin | Single protein | P81605 | US-9481662-B2 |
| Kinase | Tbio | CHEMBL4105787 | Diacylglycerol kinase alpha | Single protein | P23743 | WO-2017177037-A1 |
| Enzyme | Tbio | CHEMBL4523236 | DNA replication ATP-dependent helicase/nuclease DNA2 | Single protein | P51530 | US-9932310-B2 |
| Enzyme | Tbio | CHEMBL1250380 | Dual specificity protein phosphatase 5 | Single protein | Q16690 | US-20180000778-A1 |
| Enzyme | Tbio | CHEMBL1250381 | Dual specificity protein phosphatase 6 | Single protein | Q16828 | US-20180000778-A1  US-9127016-B2 |
| Other | Tbio | CHEMBL4523118 | Dynamin-1-like protein | Single protein | O00429 | WO-2018200674-A1 |
| Other | Tbio | CHEMBL4958 | Dynamin-1 | Single protein | Q05193 | US-20120122968-A1 |
| Enzyme | Tbio | CHEMBL4523151 | Ectonucleoside triphosphate diphosphohydrolase 5 | Single protein | O75356 | WO-2012065139-A2 |
| Other | Tbio | CHEMBL1293275 | Eyes absent homolog 2 | Single protein | O00167 | US-9725430-B2  WO-2012096977-A1 |
| Other | Tbio | CHEMBL4296245 | Eyes absent homolog 3 | Single protein | Q99504 | US-9725430-B2 |
| Enzyme | Tbio | CHEMBL4523346 | Acylpyruvase FAHD1, mitochondrial | Single protein | Q6P587 | WO-2016066849-A1 |
| Other | Tbio | CHEMBL4523304 | Fascin | Single protein | Q16658 | US-20170158692-A1  US-20140080843-A1 |
| Other | Tbio | CHEMBL4523397 | Fibrinogen C domain-containing protein 1 | Single protein | Q8N539 | US-20140120098-A1 |
| Enzyme | Tbio | CHEMBL2331065 | Alpha-ketoglutarate-dependent dioxygenase FTO | Single protein | Q9C0B1 | WO-2018157842-A1 |
| Other | Tbio | CHEMBL4523286 | GRB2-associated-binding protein 1 | Single protein | Q13480 | US-20170311597-A1 |
| Kinase | Tbio | CHEMBL1293257 | Galactokinase | Single protein | P51570 | US-8357701-B2 |
| Enzyme | Tbio | CHEMBL3588729 | Ghrelin o-acyltransferase | Single protein | Q96T53 | US-20170056373-A1  US-8329745-B2 |
| GPCR | Tbio | CHEMBL4523229 | G-protein coupled receptor 1 | Single protein | P46091 | US-8709734-B2 |
| Other | Tbio | CHEMBL3989381 | Hepcidin | Single protein | P81172 | EP-2473486-B1  US-20120196853-A1  US-20120202806-A1  US-20120214798-A1 |
| Other | Tbio | CHEMBL5878 | Histidine triad nucleotide-binding protein 1 | Single protein | P49773 | US-20180065965-A1 |
| Enzyme | Tbio | CHEMBL4523192 | Histidine decarboxylase | Single protein | P19113 | US-8207292-B2 |
| Transcription Factor | Tbio | CHEMBL2311236 | High mobility group protein B1 | Single protein | P09429 | US-9932355-B2  WO-2017142324-A2 |
| Enzyme | Tbio | CHEMBL4523419 | Serine protease HTRA1 | Single protein | Q92743 | US-20180371015-A1  WO-2016100555-A1  WO-2017222914-A1  WO-2018015240-A1  WO-2018036942-A1  WO-2018036957-A1 |
| Enzyme | Tbio | CHEMBL4523137 | Serine protease HTRA2, mitochondrial | Single protein | O43464 | US-8242122-B2 |
| Enzyme | Tbio | CHEMBL4523486 | Hydroxyproline dehydrogenase | Single protein | Q9UF12 | US-20180002275-A1  WO-2017100268-A1 |
| Enzyme | Tbio | CHEMBL3627587 | Indoleamine 2,3-dioxygenase 2 | Single protein | Q6ZQW0 | WO-2017114260-A1 |
| Other | Tbio | CHEMBL4296013 | Interleukin-23 receptor | Single protein | Q5VWK5 | US-9624268-B2 |
| Transcription Factor | Tbio | CHEMBL3407312 | Krueppel-like factor 10 | Single protein | Q13118 | US-20170121318-A1 |
| Transcription Factor | Tbio | CHEMBL3407312 | Krueppel-like factor 5 | Single protein | Q13887 | US-20150246908-A1 |
| Kinase | Tbio | CHEMBL6167 | Serine/threonine-protein kinase LATS1 | Single protein | O95835 | US-20180344738-A1 |
| Enzyme | Tbio | CHEMBL5942 | Phosphatidylcholine-sterol acyltransferase | Single protein | P04180 | EP-2862861-B1  US-20180170926-A1  US-9796709-B2 |
| Enzyme | Tbio | CHEMBL2249 | Protein-lysine 6-oxidase | Single protein | P28300 | WO-2017141049-A1 |
| Enzyme | Tbio | CHEMBL3714029 | Lysyl oxidase homolog 2 | Single protein | Q9Y4K0 | US-20170369443-A1  US-20180186755-A1  US-20180215727-A1  WO-2017139274-A1  WO-2017141049-A1  WO-2018048930-A1  WO-2018048943-A1 |
| Other | Tbio | CHEMBL2692 | Cellular retinoic acid-binding protein II | Single protein | P29373 | US-8409550-B2 |
| Enzyme | Tbio | CHEMBL3313835 | S-adenosylmethionine synthase isoform type-2 | Single protein | P31153 | US-20180079753-A1 |
| Enzyme | Tbio | CHEMBL4523324 | Mesoderm-specific transcript homolog protein | Single protein | Q5EB52 | US-20120270250-A1 |
| Other | Tbio | CHEMBL3580494 | Mucin-1 | Single protein | P15941 | US-8952054-B2 |
| Enzyme | Tbio | CHEMBL4523354 | Nicotinate phosphoribosyltransferase | Single protein | Q6XQN6 | WO-2017162840-A1 |
| Other | Tbio | CHEMBL2016430 | NEDD8 activating enzyme catalytic subunit | Single protein | Q8TBC4 | WO-2013028832-A2  WO-2017183927-A1 |
| Enzyme | Tbio | CHEMBL4523426 | Endonuclease 8-like 1 | Single protein | Q96FI4 | US-20170038365-A1 |
| Enzyme | Tbio | CHEMBL2726 | Sialidase-1 | Single protein | Q99519 | WO-2018213933-A1 |
| Enzyme | Tbio | CHEMBL3200 | Sialidase-2 | Single protein | Q9Y3R4 | WO-2018213933-A1 |
| Enzyme | Tbio | CHEMBL3046 | Sialidase-3 | Single protein | Q9UQ49 | WO-2018213933-A1 |
| Other | Tbio | CHEMBL4523246 | Neurotrypsin | Single protein | P56730 | US-20130261130-A1 |
| Enzyme | Tbio | CHEMBL3708197 | Cytosolic purine 5'-nucleotidase | Single protein | P49902 | WO-2018067834-A1 |
| Enzyme | Tbio | CHEMBL3396944 | N-glycosylase/DNA lyase | Single protein | O15527 | US-20170038365-A1 |
| oGPCR | Tbio | CHEMBL4523454 | Olfactory receptor 51E2 | Single protein | Q9H255 | US-20180116992-A1 |
| Other | Tbio | CHEMBL5922 | Multifunctional protein ADE2 | Single protein | P22234 | WO-2018122550-A1 |
| Kinase | Tbio | CHEMBL6054 | PAS domain-containing serine/threonine-protein kinase | Single protein | Q96RG2 | EP-2681207-B1  US-20120220589-A1  US-20120225846-A1  US-20120225863-A1  US-20120277224-A1  US-20140128392-A1  US-20150031679-A1  US-20150274740-A1  US-20150284395-A1 |
| Other | Tbio | CHEMBL4523490 | Phosphatidylcholine transfer protein | Single protein | Q9UKL6 | US-9556129-B2 |
| Transporter | Tbio | CHEMBL4523138 | Pendrin | Single protein | O43511 | WO-2017147523-A1 |
| Transporter | Tbio | CHEMBL4523223 | Chloride anion exchanger | Single protein | P40879 | WO-2017147523-A1 |
| Enzyme | Tbio | CHEMBL3334418 | Phosphoglycerate mutase 1 | Single protein | P18669 | US-20140294818-A1 |
| Other | Tbio | CHEMBL5962 | Phospholipid transfer protein | Single protein | P55058 | EP-2772488-A1  EP-2772488-B1 |
| Enzyme | Tbio | CHEMBL4523434 | Bifunctional polynucleotide phosphatase/kinase | Single protein | Q96T60 | US-9694073-B2 |
| Enzyme | Tbio | CHEMBL3988601 | Porphobilinogen Deaminase | Single protein | P08397 | US-20130172397-A1 |
| Enzyme | Tbio | CHEMBL4295747 | Serine hydroxymethyltransferase, mitochondrial | Single protein | P34897 | WO-2016085990-A1 |
| Other | Tbio | CHEMBL2079846 | Son of sevenless homolog 1 | Single protein | Q07889 | WO-2016077793-A1  WO-2018115380-A1 |
| Other | Tbio  Tbio | CHEMBL2095174 | SUMO activating enzyme | Protein complex | Q9UBE0 Q9UBT2 | WO-2017183927-A1 |
| Enzyme | Tbio | CHEMBL6094 | Ras-related C3 botulinum toxin substrate 1 | Single protein | P63000 | US-8884006-B2 |
| Other | Tbio | CHEMBL2362978 | DNA repair protein RAD52 homolog | Single protein | P43351 | US-20180134727-A1  US-20180209956-A1 |
| Other | Tbio | CHEMBL4295974 | Regulator of G-protein signaling 17 | Single protein | Q9UGC6 | US-20160264616-A1 |
| Enzyme | Tbio | CHEMBL6052 | Transforming protein RhoA | Single protein | P61586 | US-8884006-B2 |
| Enzyme | Tbio | CHEMBL3831182 | Retinoid isomerohydrolase | Single protein | Q16518 | US-20130225694-A1  US-20170340581-A1 |
| Enzyme | Tbio | CHEMBL6118 | Sulfide:quinone oxidoreductase, mitochondrial | Single protein | Q08257 | WO-2017123823-A1 |
| Other | Tbio | CHEMBL2362976 | Protein S100-A4 | Single protein | P26447 | US-20130090355-A1 |
| Other | Tbio | CHEMBL4296265 | Protein S100-A9 | Single protein | P06702 | US-20180155330-A1 |
| Enzyme | Tbio | CHEMBL4523507 | Deoxynucleoside triphosphate triphosphohydrolase SAMHD1 | Single protein | Q9Y3Z3 | US-20180313843-A1 |
| Other | Tbio | CHEMBL4523350 | Sterile alpha and TIR motif-containing protein 1 (in chembl: NAD(+) hydrolase SARM1) | Single protein | Q6SZW1 | WO-2018057989-A1 |
| Other | Tbio | CHEMBL3580487 | Sclerostin | Single protein | Q9BQB4 | US-20140023653-A1  US-9708375-B2 |
| Enzyme | Tbio | CHEMBL3988583 | Sepiapterin reductase | Single protein | P35270 | US-9963462-B2 |
| Enzyme | Tbio | CHEMBL4460 | Serine racemase | Single protein | Q9GZT4 | US-20140371291-A1 |
| Enzyme | Tbio | CHEMBL3879859 | E3 ubiquitin-protein ligase SMURF1 | Single protein | Q9HCE7 | EP-3143014-B1  US-20150329529-A1  US-20170088546-A1 |
| Enzyme | Tbio | CHEMBL4523460 | E3 ubiquitin-protein ligase SMURF2 | Single protein | Q9HAU4 | US-20170088546-A1 |
| Transcription Factor | Tbio | CHEMBL4523220 | Transcription factor SOX-18 | Single protein | P35713 | WO-2018112545-A1 |
| Other | Tbio | CHEMBL3399912 | StAR-related lipid transfer protein 7, mitochondrial | Single protein | Q9NQZ5 | US-9556129-B2 |
| Other | Tbio | CHEMBL4523506 | START domain-containing protein 10 | Single protein | Q9Y365 | US-9556129-B2 |
| Other | Tbio | CHEMBL4523377 | Stimulator of interferon genes protein | Single protein | Q86WV6 | US-20180258132-A1 |
| Enzyme | Tbio | CHEMBL1743291 | Sulfotransferase 1A1 | Single protein | P50225 | US-8372380-B2 |
| Epigenetic | Tbio | CHEMBL1795177 | Histone-lysine N-methyltransferase SUV39H2 | Single protein | Q9H5I1 | US-20180273529-A1 |
| Other | Tbio | CHEMBL4523136 | Synaptojanin-1 | Single protein | O43426 | US-20170042857-A1  WO-2015079413-A2 |
| Other | Tbio | CHEMBL4523129 | Synaptojanin-2 | Single protein | O15056 | US-20170042857-A1  US-20170165285-A1  WO-2015079413-A2 |
| GPCR | Tbio | CHEMBL3309106 | Taste receptor type 2 member 16 | Single protein | Q9NYV7 | EP-2137322-B1  EP-2267129-B1  EP-2494350-A1  EP-2769216-A1  US-8187822-B2  US-8673578-B2 |
| Other | Tbio | CHEMBL4523127 | Transmembrane 4 L6 family member 5 | Single protein | O14894 | US-8445722-B2 |
| Enzyme | Tbio | CHEMBL2331048 | Transmembrane protease serine 4 | Single protein | Q9NRS4 | US-20140221411-A1  WO-2013058613-A2 |
| Other | Tbio | CHEMBL3713062 | Tissue factor pathway inhibitor | Single protein | P10646 | US-20130252896-A1  US-8466108-B2 |
| Epigenetic | Tbio | CHEMBL2176772 | E3 ubiquitin-protein ligase TRIM33 | Single protein | Q9UPN9 | WO-2018200988-A1 |
| Other | Tbio | CHEMBL4295995 | Tolloid-like protein 2 | Single protein | Q9Y6L7 | US-20160340328-A1  US-20180362485-A1  WO-2017006295-A1 |
| Enzyme | Tbio | CHEMBL4295975 | Ubiquitin carboxyl-terminal hydrolase 25 | Single protein | Q9UHP3 | WO-2017139778-A1  WO-2017139779-A1 |
| Enzyme | Tbio | CHEMBL2157853 | Ubiquitin carboxyl-terminal hydrolase 28 | Single protein | Q96RU2 | WO-2017139778-A1  WO-2017139779-A1 |
| Enzyme | Tbio | CHEMBL4523357 | Ubiquitin carboxyl-terminal hydrolase 30 | Single protein | Q70CQ3 | US-20180086708-A1  WO-2017093718-A1  WO-2018060689-A1  WO-2018060742-A1  WO-2018065768-A1  WO-2018213150-A1 |
| Enzyme | Tbio | CHEMBL2406899 | Probable ubiquitin carboxyl-terminal hydrolase FAF-X | Single protein | Q93008 | US-20160237082-A1 |
| Other | Tbio | CHEMBL3334415 | Transcriptional coactivator YAP1 | Single protein | P46937 | WO-2018053446-A1 |
| GPCR | Tdark | CHEMBL3559705 | Taste receptor type 2 member 20 | Single protein | P59543 | EP-2494350-A1  EP-2769216-A1  US-8673578-B2 |
| Other | Tbio | CHEMBL3758068 | Cullin-1 | Single protein | Q13616 | WO-2014031759-A2 |
| Enzyme | Tbio | CHEMBL3351199 | Protein phosphatase 1G | Single protein | O15355 | US-20180000778-A1 |
| Kinase | Tbio | CHEMBL5969 | Phosphatidylinositol-4-phosphate 5-kinase type-1 alpha | Single protein | Q99755 | WO-2016014992-A2 |
| Transcription factor | Tbio | CHEMBL1250363 | Protein max | Single protein | P61244 | US-20140296307-A1  US-20150291521-A1  US-20160264560-A1  US-20170233405-A1  WO-2018112545-A1 |
| Transcription factor | Tbio | CHEMBL3407313 | Krueppel-like factor 1 | Single protein | Q13351 | US-20170121318-A1 |
| Enzyme | Tbio | CHEMBL3734642 | Glycerol-3-phosphate acyltransferase 1, mitochondrial | Single protein | Q9HCL2 | US-20120270250-A1 |
| GPCR | Tbio | CHEMBL3309107 | Taste receptor type 2 member 4 | Single protein | Q9NYW5 | EP-2494350-A1  EP-2769216-A1  US-8673578-B2 |
| Transcription factor | Tbio | CHEMBL5029 | Proto-oncogene protein c-fos | Single protein | P01100 | WO-2018112545-A1 |
| Other | Tbio | CHEMBL3233 | Adenomatous polyposis coli protein | Single protein | P25054 | US-8242122-B2 |
| GPCR | Tdark | CHEMBL3309110 | Taste receptor type 2 member 9 | Single protein | Q9NYW1 | EP-2494350-A1  EP-2769216-A1  US-8673578-B2 |
| Enzyme | Tbio | CHEMBL3924 | Dual specificity phosphatase 22 | Single protein | Q9NRW4 | US-20180000778-A1 |
| GPCR | Tbio | CHEMBL3559704 | Taste receptor type 2 member 10 | Single protein | Q9NYW0 | EP-2137322-B1  EP-2267129-B1  EP-2494350-A1  EP-2769216-A1  US-8187822-B2  US-8673578-B2 |
| GPCR | Tdark | CHEMBL3309109 | Taste receptor type 2 member 60 | Single protein | P59551 | EP-2494350-A1  EP-2769216-A1  US-8673578-B2 |
| Enzyme | Tbio | CHEMBL4850 | Receptor-type tyrosine-protein phosphatase epsilon | Single protein | P23469 | US-20180000778-A1 |
| GPCR | Tdark | CHEMBL3309108 | Taste receptor type 2 member 41 | Single protein | P59536 | EP-2494350-A1  EP-2769216-A1  US-20130115624-A1  US-8673578-B2 |
| Enzyme | Tbio | CHEMBL4546 | Serine/threonine-protein phosphatase PP1-beta catalytic subunit | Single protein | P62140 | US-20180000778-A1 |
| Enzyme | Tbio | CHEMBL6087 | Ras-related C3 botulinum toxin substrate 3 | Single protein | P60763 | US-8884006-B2 |
| Other | Tbio | CHEMBL4296241 | Pleckstrin homology domain-containing family A member 7 | Single protein | Q6IQ23 | US-20160304533-A1 |
| Enzyme | Tbio | CHEMBL1795098 | Carboxy-terminal domain RNA polymerase II polypeptide A small phosphatase 1 | Single protein | Q9GZU7 | WO-2012096977-A1 |
| Other | Tbio | CHEMBL3232697 | T-lymphoma invasion and metastasis-inducing protein 1 | Single protein | Q13009 | EP-2931280-B1 |
| Other | Tbio | CHEMBL3308929 | Double-strand break repair protein MRE11A | Single protein | P49959 | US-9932310-B2 |
| Transcription factor | Tbio | CHEMBL1293249 | Kruppel-like factor 5 | Single protein | Q13887 | US-20150246908-A1 |
| GPCR | Tbio | CHEMBL3559706 | Taste receptor type 2 member 50 | Single protein | P59544 | EP-2494350-A1  EP-2769216-A1  US-8673578-B2 |
| GPCR | Tbio | CHEMBL3309105 | Taste receptor type 2 member 14 | Single protein | Q9NYV8 | EP-2267129-B1  EP-2494350-A1  EP-2769216-A1  US-8673578-B2 |
| Enzyme | Tbio | CHEMBL2077 | Alcohol sulfotransferase | Single protein | Q06520 | US-8372380-B2 |
| Other | Tbio | CHEMBL4742 | Guanine nucleotide-binding protein G(o), alpha subunit 1 | Single protein | P09471 | US-20160264616-A1 |
| GPCR | Tbio | CHEMBL3714130 | G-protein coupled receptor 6 | Single protein | P46095 | EP-2882722-A1  EP-3083620-A1  EP-3083620-B1  EP-3105216-A1  WO-2018183145-A1 |
| GPCR | Tbio | CHEMBL4523249 | Taste receptor type 2 member 39 | Single protein | P59534 | EP-2323502-B1 |
| GPCR | Tbio | CHEMBL4523472 | Taste receptor type 2 member 7 | Single protein | Q9NYW3 | EP-2329271-B1 |
| Kinase | Tbio | CHEMBL4523432 | Serine/threonine-protein kinase LMTK3 | Single protein | Q96Q04 | EP-2556169-B1 |
| GPCR | Tbio | CHEMBL4523253 | Taste receptor type 2 member 46 | Single protein | P59540 | US-8603762-B2 |
| GPCR | Tdark | CHEMBL4523430 | Mas-related G-protein coupled receptor member X4 | Single protein | Q96LA9 | WO-2018232316-A1 |
