# Supplementary material for: Illuminating the druggable genome through patent bioactivity data
Source: PeerJ. 2023 May 2;11:e15153. doi: 10.7717/peerj.15153 (PMC10162037; doi:10.7717/peerj.15153)
Supplement: Supplemental Information 2 [file peerj-11-15153-s002.docx]

| **Target name** | **Uniprot ID** | **Number of compounds** |
| --- | --- | --- |
| Kinases | | |
| Serine/threonine-protein kinase LATS1 | O95835 | 184 |
| Serine/threonine-protein kinase LMTK3 | Q96Q04 | 2 |
| GPCRs | | |
| G-protein coupled receptor 6 | P46095 | 187 |
| Olfactory receptor 51E2 | Q9H255 | 13 |
| G-protein coupled receptor 1 | P46091 | 4 |
| Taste receptor type 2 member 46 | P59540 | 1 |
| Taste receptor type 2 member 20 | P59543 | 1 |
| Taste receptor type 2 member 39 | P59534 | 1 |
| Taste receptor type 2 member 41 | P59536 | 1 |
| Taste receptor type 2 member 50 | P59544 | 1 |
| Taste receptor type 2 member 60 | P59551 | 1 |
| Taste receptor type 2 member 14 | Q9NYV8 | 1 |
| Taste receptor type 2 member 10 | Q9NYW0 | 1 |
| Taste receptor type 2 member 9 | Q9NYW1 | 1 |
| Taste receptor type 2 member 4 | Q9NYW5 | 1 |
| Taste receptor type 2 member 7 | Q9NYW3 | 1 |
| Taste receptor type 2 member 16 | Q9NYV7 | 1 |
| Other | | |
| Tissue factor pathway inhibitor | P10646 | 1127 |
| Sepiapterin reductase | P35270 | 904 |
| Histone-lysine N-methyltransferase SUV39H2 | Q9H5I1 | 460 |
| Ubiquitin carboxyl-terminal hydrolase 30 | Q70CQ3 | 436 |
| Interleukin-23 receptor | Q5VWK5 | 403 |
| Son of sevenless homolog 1 | Q07889 | 214 |
| Acetyl-coenzyme A synthetase, cytoplasmic | Q9NR19 | 210 |
| Ubiquitin-like modifier-activating enzyme ATG7 | O95352 | 191 |
| Serine protease HTRA1 | Q92743 | 184 |
| D-3-phosphoglycerate dehydrogenase | O43175 | 163 |
| Lysyl oxidase homolog 2 | Q9Y4K0 | 153 |
| Phosphatidylcholine-sterol acyltransferase | P04180 | 134 |
| Ubiquitin carboxyl-terminal hydrolase 28 | Q96RU2 | 117 |
| Ubiquitin carboxyl-terminal hydrolase 25 | Q9UHP3 | 111 |
| Myc proto-oncogene protein | P01106 | 94 |
| E3 ubiquitin-protein ligase SMURF1 | Q9HCE7 | 78 |
| 7-alpha-hydroxycholest-4-en-3-one 12-alpha-hydroxylase | Q9UNU6 | 77 |
| Cyclic GMP-AMP synthase | Q8N884 | 57 |
| S-adenosylmethionine synthase isoform type-2 | P31153 | 56 |
| Multifunctional protein ADE2 | P22234 | 38 |
| 2-amino-3-carboxymuconate-6-semialdehyde decarboxylase | Q8TDX5 | 28 |
| Exportin-1 | O14980 | 24 |
| Nicotinate phosphoribosyltransferase | Q6XQN6 | 22 |
| Alpha-ketoglutarate-dependent dioxygenase FTO | Q9C0B1 | 21 |
| Protein S100-A9 | P06702 | 17 |
| Integrin alpha-5/beta-1 | P05556/  P08648 | 16 |
| E3 ubiquitin-protein ligase TRIM33 | Q9UPN9 | 16 |
| Sclerostin | Q9BQB4 | 16 |
| Transcriptional coactivator YAP1 | P46937 | 12 |
| Neurotrypsin | P56730 | 11 |
| SUMO-activating enzyme | Q9UBE0/  Q9UBT2 | 11 |
| DNA repair protein RAD52 homolog | P43351 | 8 |
| Sialidase 1 | Q99519 | 8 |
| Fascin | Q16658 | 8 |
| Cysteine protease ATG4B | Q9Y4P1 | 7 |
| N-glycosylase/DNA lyase | O15527 | 7 |
| B-cell lymphoma 3 protein | P20749 | 6 |
| Serine hydroxymethyltransferase, mitochondrial | P34897 | 6 |
| E3 ubiquitin-protein ligase SMURF2 | Q9HAU4 | 5 |
| DEP domain-containing mTOR-interacting protein | Q8TB45 | 5 |
| Sialidase 3 | Q9UQ49 | 5 |
| Disabled homolog 2-interacting protein | Q5VWQ8 | 4 |
| Cytosolic carboxypeptidase 2 | Q5U5Z8 | 4 |
| Stimulator of interferon genes protein | Q86WV6 | 4 |
| Histidine triad nucleotide-binding protein 1 | P49773 | 4 |
| Sulfotransferase 1A1 | P50225 | 4 |
| Kruppel-like factor 5 | Q13887 | 3 |
| Cholesterol 24-hydroxylase | Q9Y6A2 | 3 |
| NAD(+) hydrolase SARM1 | Q6SZW1 | 2 |
| Dynamin-1-like protein | O00429 | 2 |
| GRB2-associated-binding protein 1 | Q13480 | 2 |
| Dual specificity protein phosphatase 5 | Q16690 | 1 |
| Regulator of G-protein signaling 17 | Q9UGC6 | 1 |
| Indoleamine 2,3-dioxygenase 2 | Q6ZQW0 | 1 |
| Dual specificity protein phosphatase 6 | Q16828 | 1 |
| Aldehyde oxidase | Q06278 | 1 |
| Protein S100-A4 | P26447 | 1 |
| Synaptojanin-2 | O15056 | 1 |
| Mucin-1 | P15941 | 1 |
