# Supplementary material for: Illuminating the druggable genome through patent bioactivity data
Source: PeerJ. 2023 May 2;11:e15153. doi: 10.7717/peerj.15153 (PMC10162037; doi:10.7717/peerj.15153)
Supplement: Supplemental Information 3 [file peerj-11-15153-s003.docx]

| **IDG target class** | **N. of targets** | **N. of patents** | **N. of total compounds** | **N. of compouds within cut-off** |
| --- | --- | --- | --- | --- |
| Enzyme | 63 | 80 | 4889 | 2480 |
| Epigenetic | 3 | 3 | 882 | 476 |
| GPCR | 17 | 19 | 910 | 207 |
| Kinase | 6 | 14 | 1063 | 186 |
| Transcription factor | 10 | 15 | 1087 | 81 |
| Transporter | 2 | 1 | 23 | 0 |
| Other | 54 | 63 | 4968 | 2036 |
